# Temporal Stability of GPS Transmitter Group Delay Variations

**DOI:** 10.3390/s18061744

**Published:** 2018-05-29

**Authors:** Susanne Beer, Lambert Wanninger

**Affiliations:** Geodätisches Institut, Technische Universität Dresden, 01062 Dresden, Germany; lambert.wanninger@tu-dresden.de

**Keywords:** GPS satellite antennas, code pseudorange, group delay variations, multipath combination, SVN55

## Abstract

The code observable of global navigation satellite systems (GNSS) is influenced by group delay variations (GDV) of transmitter and receiver antennas. For the Global Positioning System (GPS), the variations can sum up to 1 m in the ionosphere-free linear combination and thus can significantly affect precise code applications. The contribution of the GPS transmitters can amount to 0.8 m peak-to-peak over the entire nadir angle range. To verify the assumption of their time-invariance, we determined daily individual satellite GDV for GPS transmitter antennas over a period of more than two years. Dual-frequency observations of globally distributed reference stations and their multipath combination form the basis for our analysis. The resulting GPS GDV are stable on the level of a few centimeters for C1, P2, and for the ionosphere-free linear combination. Our study reveals that the inconsistencies of the GDV of space vehicle number (SVN) 55 with respect to earlier studies are not caused by temporal instabilities, but are rather related to receiver properties.

## 1. Introduction

Global navigation satellite system (GNSS) transmitter and receiver antennas cause group delay variations (GDV) which affect the pseudorange observable. GDV are frequency-dependent and vary with nadir angle of the transmitted signal and with the elevation of the received signal. In the case of GPS (Global Positioning System) transmitters, space vehicle number (SVN) 49 exhibits the most pronounced GDV with a peak-to-peak difference of several meters in the ionosphere-free linear combination (IF). The main contribution to the GDV comes from the L1 signal. The exceptionally high GDV of SVN49 stems from internal signal reflections of L1 and L2 at the auxiliary L5 port, resulting in a multipath signal superimposed on the primary signal [1,2,3]. Springer and Dilssner [4] took a detailed look at this phenomenon by analyzing pseudorange residuals of IGS (International GNSS Service) stations on Earth from the precise orbit determination (POD) of May 2009. They identified further GPS Block IIR and IIR-M satellites with similar anomalies as SVN49 but much smaller in size, i.e., up to 1 m peak-to-peak in the IF. As stated in Reference [4], these satellites carry a classified payload connected to the same auxiliary port as the L5 signal on SVN49, but include a component to negate or dampen the secondary-path signal.

Haines et al. [5,6,7] determined GDV for GPS based on post-fit POD residuals from more than five years of observations of one of the two low earth orbiting (LEO) GRACE (Gravity Recovery and Climate Experiment) satellites. Their findings agree with Springer and Dilssner [4]. The largest and most differing GDV occur for GPS Block IIR and IIR-M antennas while the GDV of the Block IIA transmitters are uniform and smaller, i.e., 10 cm peak-to-peak in the IF of L1 and L2.

For kinematic POD based on raw GPS observations, Zehentner [8] combined data from more than ten LEO satellites, covering a timespan of 13 years, and estimated azimuth and elevation-dependent GDV for P1 and P2 for individual satellites. Their peak-to-peak differences reach up to around 20 cm for P1 and 10 cm for P2. Aside from internal multipath signals as in the case of SVN49, GPS transmitter GDV are also attributed to an imperfect balancing of the individual antenna elements. Since the antenna elements are arranged in circles, the GDV are supposed to only be nadir-dependent [9]. However, the P2 patterns of Zehentner [8] show some azimuth-dependencies for Block IIR-A, IIR-B, and IIF satellites.

Wanninger et al. [10] calibrated C1 and P2 GDV for GPS transmitter and receiver antennas with respect to dual-frequency carrier phase observations during one week in May 2015. Their GPS satellite GDV refer to a set of four receiving antenna types of 43 globally distributed reference stations. Despite different approaches, these results differ by less than 10 cm root mean square (RMS) from the findings of Haines et al. [7] and Zehentner [8] for the IF. The differences from Zehentner [8] for C1/P1 and P2 are around 3 cm RMS. Combined satellite and receiver antenna GDV reach some decimeters in C1 and P2 and 1 m in the IF. Their correction improves the height component by several centimeters in single-frequency precise point positioning (PPP) based on the ionosphere-free code phase combination, enhances ambiguity fixing with the Melbourne–Wübbena (MW) linear combination, and improves the TEC (total electron content) determination based on code observables.

**Figure 1:**
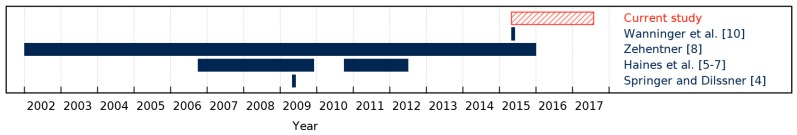
Timespans over which GPS transmitter group delay variations have been estimated. Full blue bars indicate time-independent estimations (Wanninger et al. [10], Zehentner [8], Haines et al. [5,6,7], Springer and Dilssner [4]). The striped red bar marks the temporal extent of the current study.

Induced by physical or electronic properties of the satellites, the GPS transmitter GDV are considered to be time-invariant and as far as we know, all of the aforementioned studies estimated GDV models for the complete timespans of their respective studies, as illustrated in Figure 1 by the full blue bars. Since GDV can be used as corrections for code pseudorange observations, we focus on their long-term validity. The aim of this work is to get detailed insight in the temporal behavior of the GPS GDV. Therefore, we estimate daily individual satellite GDV based on the approach of References [10,11]. We measure temporal variations using daily RMS with respect to an overall mean GDV model.

The paper is organized as follows. In Section 2, we describe the applied methods and underlying data. Section 3 presents our results, compares them to earlier studies, and gives a detailed examination of the inconsistencies of the GDV for SVN55, with respect to earlier studies. Finally, Section 4 summarizes our main conclusions.

Throughout the paper we denote C/A code observables on L1 as C1, and P or Y code observables on L2 as P2, following the Receiver Independent Exchange Format (RINEX) 2 conventions [12]. Since we focus on the GDV of the GPS transmitters, we refer GDV to the nadir angle at the GPS satellite antenna. The term nadir angle is used synonymously with boresight angle.

## 2. Methods and Data

### 2.1. Multipath Combination and Piecewise Linear Modelling

Information on the group delays of GNSS signals can be retrieved from the so called multipath (MP) combination (Rocken and Meertens [13]), which is the difference between code and carrier phase, corrected for ionospheric delays:

(1) $$MP_{i}=C_{i}-\Phi_{i}+2\lambda_{i}^{2}\frac{\Phi_{j}-\Phi_{i}}{\lambda_{j}^{2}-\lambda_{i}^{2}}-B_{i}.$$

The characteristics of Equation (1) are well described by Simsky [14]. C and Φ denote the code and carrier phase measurements, respectively, in units of meters, i and j are the involved frequency bands, and $\lambda_{i}$ and $\lambda_{j}$ are their respective wavelengths in units of meters. $MP_{i}$ is free from geometric, ionospheric, and tropospheric contributions, but contains an arbitrary offset, $B_{i}$, due to phase ambiguities and hardware and software-induced delays. These biases cannot be separated from each other, but they are considered to be constant in continuous ambiguity sequences. Thus, $MP_{i}$ only provides relative variations within those sequences, but no absolute values. Since we combine many MP sequences, we take the various biases, $B_{i}$, into account and apply an overall zero-mean condition.

**Figure 2:**
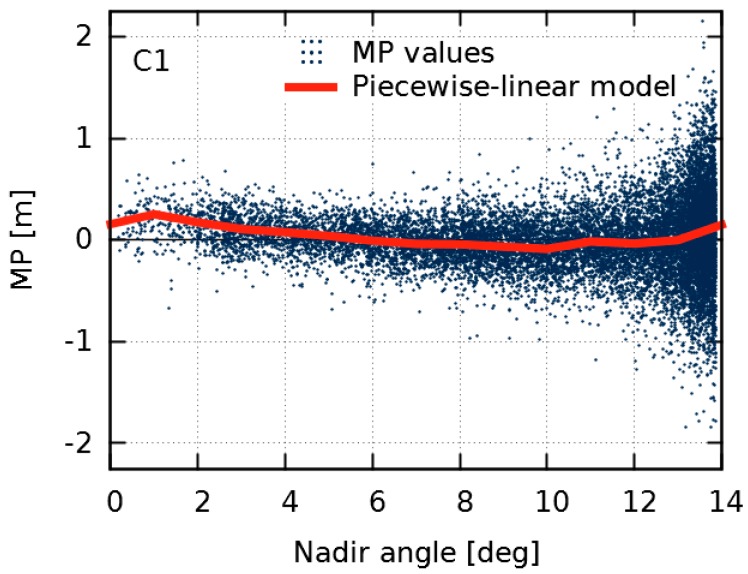
Multipath (MP) combination for GPS space vehicle number 43 for C1 (observation data of 3 May 2015 from 17 globally distributed reference stations, see Figure 3). Blue dots indicate single MP values. The red line shows the estimated piecewise linear model in steps of 1°.

**Figure 3:**
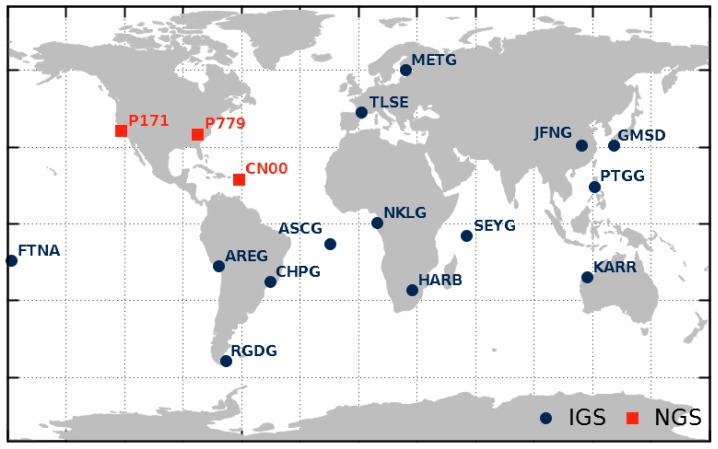
Set of 17 reference stations of the International GNSS Service (IGS, blue dots) and National Geodetic Survey (NGS, red squares). All stations are equipped with a TRM59800.00 antenna and a TRIMBLE NETR9 receiver.

High-frequency code multipath and tracking noise dominate the GDV time series. However, we are interested in the low-frequency variations. To extract them, we estimate a piecewise linear model as a function of the nadir angle in steps of 1°. Nadir angle, η, of the transmitted signal at the satellite can be calculated from elevation, e, of the received signal on Earth by:

(2) $$\sin\eta =\frac{R}{A}\cos e$$

(Schmid and Rothacher [15]). In Equation (2), R is the Earth’s radius and A is the distance between the geocenter and the satellite, which is identical to the semi-major axis of the quasi-circular GPS orbit. For GPS and stations on the Earth’s surface, nadir angles range from 0° to nearly 14° corresponding to elevations between 90° and 0°. Due to a higher noise level of the MP values at low elevations, an elevation-dependent weighting scheme is applied. Figure 2 shows a typical example of MP values and the estimated piecewise linear model for a single GPS satellite, tracked by the reference stations shown in Figure 3. We obtain most observations and MP values at high nadir angles corresponding to low elevations. For nadir angles close to 0° there are comparatively few observations, since they can only be obtained when the satellite passes a station in zenith; when the station is located on the satellite’s ground track. Therefore, in our individual satellite analysis, there are always several stations which provide observations at low elevations/high nadir angles and only a few stations which provide observations at high elevations/low nadir angles.

### 2.2. Data Basis

Since the ground tracks of the GPS constellation do not usually change, we aimed for a numerous set of globally distributed reference stations to get observations in the entire nadir angle range for each satellite, which also reduced site-specific multipath by averaging. We performed a daily analysis of observation data for the 17 stations shown in Figure 3 over a period of 26 month (May 2015–July 2017).

Because transmitter and receiver GDV cannot be separated, we chose stations with identical antenna and receiver types (TRM59800.00 antennas and TRIMBLE NETR9 receivers). Thus, the influence of receiver antenna and receiver type is considered to be identical for each satellite during the entire period of investigation. This makes the individual satellite GDV comparable and changes can be more easily attributed to the transmitters. However, we had to use mixed receiver firmware versions, as well as station antennas with and without domes. Further requirements for the stations were that the elevation mask must be 5° or lower, and that the site-specific multipath level must be less than 0.5 m RMS for C1 and P2 between 10° and 90° elevation.

In order to obtain GDV with centimeter accuracy, we applied absolute International GNSS Service (IGS) antenna corrections for the carrier phase observations at the receiving antenna (Dow et al. [16]) and corrections for phase wind-up effects due to satellite rotations (Wu et al. [17]). Additionally, the frequency-specific carrier phase z-offset corrections of Wanninger et al. [10] were applied for GPS satellites, since the IF values published by the IGS [16] are not suitable for our GDV determination for C1 and P2. The observation data were checked for cycle slips. Sequences spanning less than 10° of elevation angle were eliminated (approximately 10% of the observation data).

On the basis of the corrected MP values, we estimated daily satellite-specific GDV as piecewise linear functions of the nadir angle and mean GDV over the entire timespan for C1, P2, and their IF. Days with missing observations on more than five stations or with no observations below 2° nadir angle were ignored.

## 3. Results and Discussion

### 3.1. Temporal Analysis

**Figure 4:**
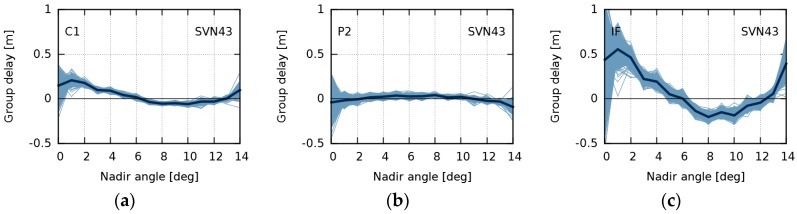
Group delay variations for space vehicle number (SVN) 43 for C1 (**a**); P2 (**b**); and the ionosphere-free (IF) linear combination (**c**). Thin light blue lines indicate daily estimations (May 2015–July 2017). The thick dark blue line indicates the mean over the entire timespan.

**Figure 5:**
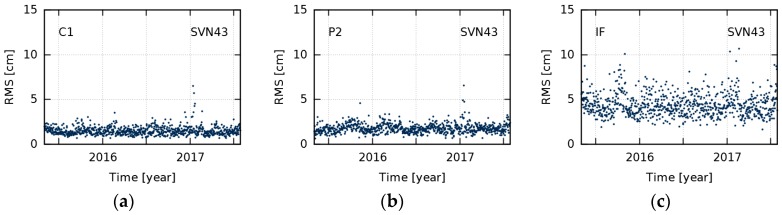
Root mean square (RMS) of differences between daily group delay variations and the overall mean for space vehicle number (SVN) 43 for C1 (**a**); P2 (**b**); and the ionosphere-free (IF) linear combination (**c**).

Daily estimates of nadir-dependent GDV were produced for every GPS satellite. Figure 4 shows daily and mean GDV models over the entire timespan for SVN43, a representative example of all the studied satellites. Due to few observations below 2° and a high noise level of observations above 13°, the daily models show a broader scatter in these peripheral nadir angle ranges. Since this scatter does not reflect temporal variations, the GDV model values of 0°, 1°, and 14° were excluded from our calculation of the daily RMS with respect to the mean. The daily RMS are used as a measure for temporal stability (Figure 5).

**Figure 6:**
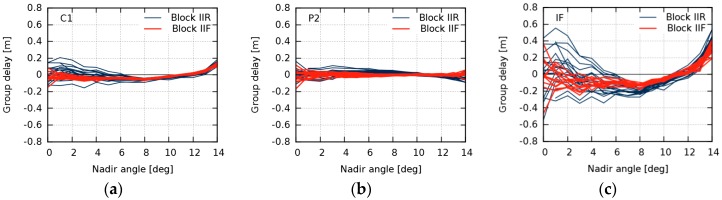
Mean group delay variations of GPS Block IIR (blue lines) and Block IIF satellites (red lines) for C1 (**a**); P2 (**b**); and the ionosphere-free (IF) linear combination (**c**).

Figure 6 shows mean GDV for all GPS satellites. The peak-to-peak variations amount to 25 cm, 14 cm, and 70 cm for C1, P2, and IF, respectively. These numbers agree well with References [8,10]. Furthermore, as already shown in References [7,10], GPS GDV are more pronounced for Block IIR satellites than for Block IIF satellites.

**Figure 7:**
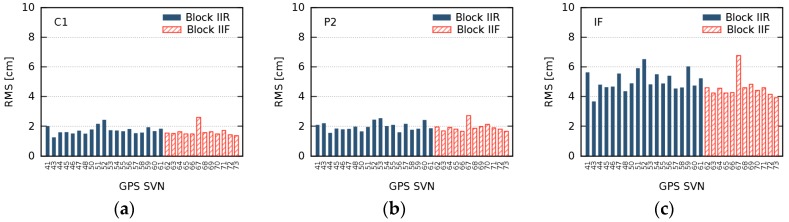
Averaged root mean square (RMS) of differences between daily and mean group delay variations for C1 (**a**); P2 (**b**); and the ionosphere-free (IF) linear combination (**c**) in the nadir angle range 2–13°. Full blue bars indicate GPS Block IIR satellites. Striped red bars indicate GPS Block IIF satellites.

Figure 7 illustrates our main results concerning the long-term behavior of GPS GDV. The RMS of differences between daily and mean GDV (as shown in Figure 5) are averaged and represent the level of temporal stability for each satellite. During the 26 months analyzed, the GPS GDV are stable on the level of approximately 2 cm for C1 and P2, and about 5 cm for the IF. At the same time, this seems to be the GDV accuracy level our approach is able to achieve. The slightly higher RMS values for few satellites, for example SVN67, may be caused by a smaller number of observations at low nadir angles, due to the uneven distribution of the reference stations.

### 3.2. Comparison to Earlier Studies and SVN55

**Figure 8:**
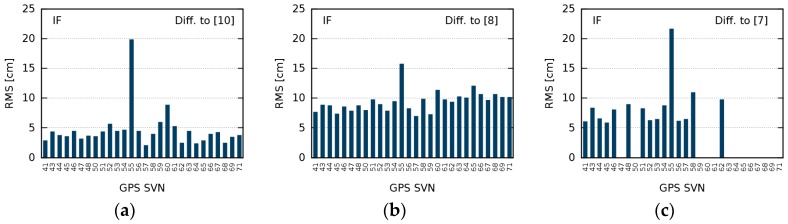
Root mean square (RMS) of differences between the group delay variations of this study and (**a**) Wanninger et al. [10]; (**b**) Zehentner [8]; and (**c**) Haines et al. [7].

In this subsection, we compare our mean GDV for the IF to earlier studies. Differences can indicate temporal GDV variations between the different time periods studied (cf. Figure 1). Since the GDV estimation of Wanninger et al. [10] refers to the start date of the current study and is also based on MP values of globally distributed terrestrial reference stations, we expected the smallest differences here. This is confirmed by differences of just around 5 cm RMS (Figure 8a). Differences to GDV of Zehentner [8] and Haines et al. [7] are larger and amount to approximately 10 cm RMS (Figure 8b,c), which can be explained by their completely different approach to determine GDV.

There is one satellite whose GDV exhibit larger differences: SVN55. Its GDV deviate by up to 20 cm RMS compared to the other studies, which even partly overlap in time with this study. Since there was no temporal variation or discontinuity detectable in the 26-month time series of this study, we conclude that the reason for the inconsistency of SVN55 must be found elsewhere. The influence of site-specific multipath, different mathematical functions in GDV modeling, the total number of observations, and the distribution of the observations in the nadir angle bins were excluded by tests.

Hauschild et al. [2] and Hauschild and Montenbruck [18,19] describe pseudorange variations and biases depending on receiver-individual multipath mitigation techniques based on correlator spacing. According to References [18,19,20], the TRIMBLE NETR9 receiver can be operated with multipath signal rejection. Although one would expect that multiple satellites would be affected by the receiver settings, we checked if the receiver model could be the reason for the GDV differences of SVN55. While we used 17 reference stations equipped with a TRIMBLE NETR9 receiver in the current study, Wanninger et al. [10] used a mixed set of 43 reference stations with only three TRIMBLE NETR9 receivers. The receiver types onboard the LEO satellites used in Zehentner [8] and Haines et al. [7] are also different.

**Figure 9:**
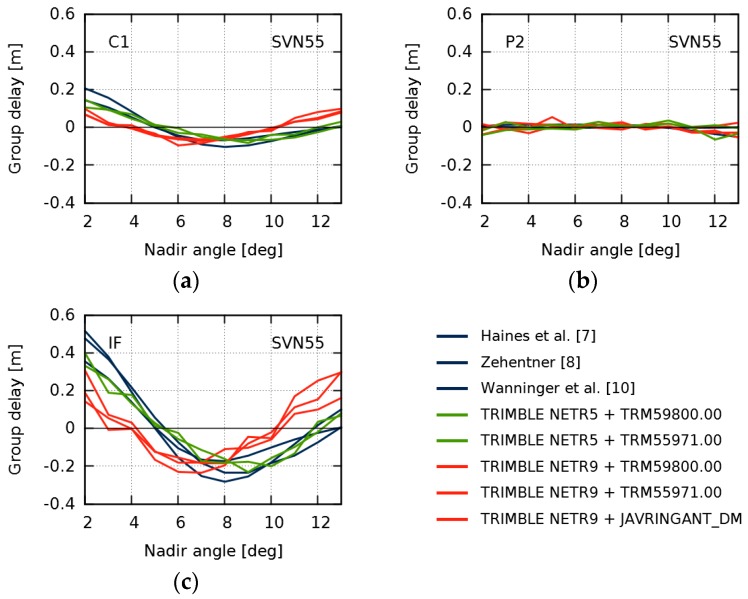
Group delay variations for GPS space vehicle number (SVN) 55 for C1 (**a**); P2 (**b**); and the ionosphere-free (IF) linear combination (**c**). Blue lines indicate results from earlier studies. Red and green lines indicate current results obtained by observations of TRIMBLE NETR9 and TRIMBLE NETR5 receivers, respectively.

Using selected stations of several additional networks (Geoscience Australia, EUREF Permanent GNSS Network (EPN), IGN Réseau GNSS Permanent, Universitary NAVSTAR Consortium (UNAVCO), African Geodetic Reference Frame (AFREF), and the TrigNet continuously operating GNSS network), we identified four more sets of reference stations with identical antenna and receiver equipment, providing observations for GPS SVN55 in the entire nadir angle range. Their GDV for SVN55 are shown in Figure 9 together with the results of the current and the earlier studies colored according to the receiver types. The results of the earlier studies and those based on observations of TRIMBLE NETR5 receivers show similar curves and their differences do not exceed 10 cm RMS in the IF. However, all results based on observations of TRIMBLE NETR9 receivers show a common different behavior with differences around 20 cm RMS when compared to TRIMBLE NETR5 and earlier results. These IF differences are mainly caused by C1 and do not depend on the receiving antenna type.

The GDV of SVN55 are those with the largest peak-to-peak variations in the earlier studies based on different receiver types. We speculate that on the stations used in the current study, the multipath rejection technique of the TRIMBLE NETR9 receivers affects satellite-induced GDV, which cannot be distinguished from site-specific multipath. However, we have no idea why this seems to affect only SVN55 and none of the other satellites with large GDV.

## 4. Conclusions

For a period of more than two years, we estimated daily GDV for 31 GPS Block IIR and Block IIF transmitter antennas based on dual-frequency observations of globally distributed reference stations. The individual satellite GDV are stable on the level of 2 cm RMS for C1 and P2, and 5 cm RMS in their IF. In comparison to some earlier studies, RMS values of the GDV differences are larger by a factor of two, which can be attributed to the very different determination method used here, but this does not indicate any temporal instabilities.

We obtained exceptional results for SVN55 whose GDV differences with respect to the other studies significantly exceed those of the other satellites. The analysis of further data sets revealed a receiver-dependency. All stations used in the current study are equipped with TRIMBLE NETR9 receivers which seem to introduce a receiver-dependent bias.
